# How to optimize communication about animal and animal-free research methods

**DOI:** 10.3389/fvets.2024.1303744

**Published:** 2024-05-28

**Authors:** Judith Regina Homberg, Aldert H. Piersma, Cyrille A. M. Krul, Lisa Genzel, Anne Kienhuis, Ellen ter Gast, Monique Wolvekamp

**Affiliations:** ^1^Radboud University Medical Center, Donders Institute for Brain, Cognition, and Behaviour, Nijmegen, Netherlands; ^2^Centre for Health Protection, RIVM, Bilthoven, Netherlands; ^3^Institute for Risk Assessment Sciences, Utrecht University, Utrecht, Netherlands; ^4^Innovative Testing in Life Sciences and Chemistry, Utrecht University of Applied Sciences, Utrecht, Netherlands; ^5^Donders Institute for Brain, Cognition, and Behaviour, Radboud University, Nijmegen, Netherlands; ^6^Consultant, Amsterdam, Netherlands

**Keywords:** animal research, animal-free methods, media, communication, stakeholders, good scientific research, mindset

## Abstract

There is a long-standing debate about experimental non-human animals and animal-free methods in scientific research. Among the various stakeholders involved in the debate are the scientists. During media broadcasts we, animal researchers and animal-free methods researchers, were positioned as ‘opponents’. In this essay we describe our initial rational thoughts and emotions after these events, and how we came together to explore our common ground on animal(−free) experimentation. Realizing that all models have advantages and limitations, our common ground lies in the principles of good scientific research and responsible experimentation. Our communication emanating from the broadcasts has been instrumental in improving communication on animal(−free) experimentation issues by teaming up. We strongly believe that this is essential for making well-informed decisions for the methods we are using now and will be using in the future.

## Introduction of people

**Judith Homberg:** Judith Homberg is Professor with a chair in Translational Neuroscience at the Radboud University Medical Center, in Nijmegen, The Netherlands. She has over 20 years of experience in research with non-human animals (mice and rats). She is using animal models to understand individual differences in behavior and cognition in the context of vulnerability and resilience to stress-related and neurodevelopmental disorders, and has a specific focus on the development of methods and tools to increase animal welfare.

**Aldert Piersma:** Aldert Piersma is Professor of Reproductive Toxicology at the Institute for Risk Assessment Sciences (IRAS) at Utrecht University. He is employed at the National Institute for Public Health and the Environment (RIVM) in Bilthoven, the Netherlands. He has been working for 30+ years on the innovation of methodologies for assessing safety and risk of chemicals and drugs, with specific attention to fertility, reproduction, pregnancy and embryo-fetal development in man. He worked in the past with mice and rats. More recently his research focuses on the development of human-relevant animal-free methods.

**Cyrille Krul:** Cyrille Krul is Professor in Innovative Testing in Life Sciences & Chemistry and Director of the Research Centre Healthy and Sustainable Living at the Utrecht University of Applied Sciences, The Netherlands. She started her scientific career in Toxicology, especially genetic and regulatory toxicology and worked initially with mice and rats. Since animals were not suitable for her research questions, she switched 25 years ago to the development and implementation of animal-free models that can be used to assess the safety of pharmaceuticals, chemicals and food ingredients. In addition, Krul trains (upcoming) professionals in advanced *in vitro* and *in silico* methods and educates people in interdisciplinary teams to accelerate the transition to animal free innovations.

### The debate on animal use in science and in the media

Animal research is a heavily debated topic. Animals are living creatures with their own intrinsic value. In research, non-human animals are used by humans to obtain knowledge about biomedical issues or to test the safety of agents that humans are being exposed to. On the one hand, these animals cannot choose to participate in experiments, they may suffer in experiments, and not all findings obtained through animal research in applied research translate to humans. On the other hand, animal research has been instrumental in developing many of the medicines and several of the biomedical tools that currently exist. This is because they sometimes gave us fundamental knowledge that was the basis for the new developments (e.g., insulin to treat diabetes, immune therapy to treat cancer, deep brain stimulation to treat Parkinson’s disease). In addition, animal studies are sometimes needed by law to get approval to start Phase 1 clinical trials or to protect human health and the environment from hazardous effects of chemicals. The friction between the first and the latter leads to debates among and between stakeholders, such as researchers, politicians and society at large. The discussion is heavily loaded with rational as well as emotional arguments. Key stakeholders in the debate are the researchers, as they have both knowledge of the scientific possibilities and progress regarding the animal(−free) research methods for biomedical and safety research, as well as the hands-on experience with the methods. While researchers are being trained to think rationally and in a fact-based manner, they are prone to using emotional arguments too. This for instance occurs in communication about animal research via the media. The understandable mix of rational and emotional arguments even within stakeholder groups on animal research complicates the debate and hampers the best-informed strategic decisions for the future. In this essay we share our individual experiences with some joint media communication about animal research and animal-free methods and how we used the confrontations staged by the media to discuss a common ground for communication on animal experimentation and animal free innovations. With that we would like to set the stage for an improvement of the discussions about animal(−free) research.

### Personal flavors on the two broadcasts that brought us together

**Judith**: On April 7th 2021 I was approached by a journalist from the Dutch television programme ‘EenVandaag” with the question if I would be available for an item in the framework of World Laboratory Animal Day. The reason for contacting me was an article in *Current Biology* (1) and related to that a published letter in ‘De Volkskrant” about animal research as unavoidable for major biomedical issues such as the COVID-19 pandemic. This letter was signed by 100 researchers and was led by me and a colleague. The contrast between the ambition of the government on the one hand and the continued need for animal research on the other hand, sometimes even because it is required by law, intrigued the journalist. The journalist was looking for someone who could explain this situation in lay language and in a nuanced manner. I said ‘yes’ to the invitation, because I considered it as a valuable opportunity to explain why animal research is still needed; this is because we do not yet have animal-free methods for all research questions and stopping animal research now would leave several very serious biomedical issues unsolved. I also wanted to explain that animal research is conducted according to very strict rules. For instance, it is not allowed if the research can be conducted without the use of animals. Finally, I wanted to make the point that we care a lot about the animals and preferably would not use animals if we would have the possibility to address our research questions through other methods. We are also putting a lot of effort into developing methods that help to improve animal welfare. The journalist indicated upfront that she was still looking for another person who has the opinion that animals should not be used in research. Via the communication officer of the Radboud University Medical Center I learned that the other person would be Aldert Piersma from the National Institute for Public Health and the Environment (RIVM) in Bilthoven, the Netherlands. However, the actual set-up of the interview with me and Piersma remained unclear. Via the web and looking at publications I got an impression of the work of Piersma. Since he is from a different research field (toxicology) compared to mine (neuroscience), I did not see an opposition. In the field of toxicology there are (more) possibilities for animal-free research approaches, and I fully agree that animals should not be used if the research questions can also be addressed without them. In the field of neuroscience, where we focus on behavior, cognition and associated brain functions, cell-based systems, for example, do not work as they cannot mimic behavior and cognition. During the recording of the interview the focus was on the De Volkskrant letter and the reasons why animal research is still being done. No link was made to the recording of Piersma. Only during the broadcast could I see the final result (2). I liked it, although some opposition between the animal-free and animal research could be felt; using animals came across less ‘moral’ compared to the use of animal-free innovations. I received overall positive feedback, but also received a few negative attacking emails from people who did not agree with animal research. I learned that there is interest to hear more about animal research but that it is also a very sensitive topic requiring careful framing and picking the right words to convey the message.

After this event I was approached on June 1 2021 by a journalist from the Dutch Radio Programme ‘Op1” with the question if I was willing to give feedback on a recently awarded project “Virtual Human Platform for safety assessment’ as a researcher using animals. I accepted the invite, as the project is really great, innovative and a step forward in the development of animal-free methods, but does not take away the need for animal research for research questions for which no animal-free methods exist yet. I considered it as another opportunity to explain that we prefer animal-free research if we would have the choice, but that with the lack of animal-free methods for several research questions animal research is still being conducted. At one moment I was invited, at the next moment I was already on the phone, live in the radio programme, to answer questions [3]. There was no direct contact with the researchers of the project. I just learned from feedback from the communication officer of our academic hospital that I largely responded as we had trained, with some minor suggestions on how to improve for a next time whilst communicating about animal research.

**Aldert Piersma:** In early April 2021 I was invited by journalists to contribute to a television broadcast item on the occasion of the annual World Day for Laboratory Animals (April 24). The hosting television show was 1 Vandaag, an informative program by the Dutch national broadcast organization (NPO) giving background to current issues. The item was focused on the replacement of animal testing by animal-free methods. After consultation with colleagues, I decided to participate for several reasons.

First, the development of animal-free methods for human chemical and pharmaceutical risk assessment is my research area of primary interest, foremostly based on the notion that animal studies are not perfect predictors for human risk, and promising animal-free methods are emerging. In addition, the ethical implications of spending many animal lives for human safety plays a significant role.

Second, as an employee of RIVM, the Dutch National Institute for Public Health and the Environment, I considered it important to contribute to the general discussion on this subject. RIVM is an important player in the Dutch stakeholder network for transition to animal-free innovations (TPI), and considers it a responsibility to stand transparently amidst the society at large, speaking out on topics of societal concern on the basis of current scientific knowledge and technology.

Third, from the scientific perspective, I saw the opportunity to draw attention to innovative New Approach Methodologies, including a multitude of cell and tissue culture methods and computational methods, combined to build a computational virtual human as a tool that is expected to revolutionize many areas of human health protection, including clinical science and chemical safety.

Fourth, in more general terms, I would like to spread the idea that in whatever area of research, also in areas where animal-free methods may not be on the horizon yet, an open mindset of researchers is needed toward opportunities for animal-free methods. Thus, while appreciating that currently animal methods may still be needed, opportunities for alternative approaches should actively be explored in every area of research that uses animal testing.

Finally, I would like to take the opportunity to stress that good science should always be the gold standard for any methodological choices. Therefore, any method, animal or animal-free, should be considered with care as to its fitness-for-purpose to answer the scientific research question at hand. In the area of chemical safety, we have to recognize that any change in methodology will by definition result in loss of certain information on the one hand and gain of different information on the other. It is the balance between the two and the enhanced relevance for human risk assessment of new approaches that will need to drive decisions on the adoption of novel, animal-free methodology.

To my satisfaction, the broadcast effectively expressed some of my messages (2). To my surprise, Judith Homberg of Nijmegen University also appeared on the show. As this was not *a priori* shared with me, we did not have the chance to communicate directly before or during the recording. In the broadcast, Judith advocated the current necessity for animal use in behavioral studies. Obviously, the program was looking for opposing positions in the discussion on replacement of animal studies. In my perception however, whereas Judith and myself spoke from different perspectives respectively, there was no disagreement between us on the necessity for good science as the driving force for methodological choices. Also, although not explicitly addressed, I felt that the need to continuously critically review current methodology and consider possibly advantageous alternatives, both from the scientific and ethical perspective, was shared between both of us. All in all, I found the broadcast worthwhile, informative, interesting, and topical.

**Cyrille:** On June 1, 2021 I was approached by a journalist of the Dutch Radio Programme ‘Op1’ with the question whether I could explain something about animal-free research. The reason was the kick-off of a new NWA project named ‘Virtual Human Platform for safety assessment’ (VHP4Safety). I agreed to give an interview, because we would like to inform the general public that a large project was granted that would not focus on the development of *in vitro* and *in silico* models only. For the first time, we have started a project that will take into account the whole spectrum from model development toward regulatory acceptance and combine many different disciplines from ICT, life sciences to social sciences. It will address not only scientific needs (better predictive models), but also industrial and societal needs. Reaching out to the general public fits within this approach. Furthermore, in the project we focus on issues that cannot or are hardly addressed with animal research. In VHP4Safety, we take the human as ‘gold standard’ instead of the animal to demonstrate that we do not want to replace animal research, but make animal research redundant.

My intention was to focus on new opportunities and on the possibilities for doing better risk assessment, instead of comparison with the current situations and *in vivo* animal studies. I prefer not to be defensive or provocative, or to oversell the new possibilities. We would like to give insights into new developments, reach out to everyone to join and try to make a difference. I am convinced that we join forces, instead of showing differences between domains and researchers. Together we can show what is (not) possible/will (not) work in other domains and how we should improve our animal-free methods. Every model has its own advantages and limitations and it is difficult to compare.

The filming and interview on the same day was quite relaxed. Very quick, because it was recorded less than 2 h before broadcasting. I was not able to listen when it was broadcasted, but I listened afterwards (3).

When I watched the episode, I was a bit surprised to learn that Judith was asked to respond. She was the representative of people that recently published a letter in De Volkskrant, one of the leading newspapers in the Netherlands, that fueled the discussion and referred to policy statements made by the NCad (Netherlands National Committee for the protection of animals used for scientific purposes). The authors of the letter perceived that the goal of the Dutch policy was to phase out *all* animal experiments by 2025, but the original statements of the NCad and the Dutch government were more nuanced and were meant as an initial target number. In the same letter the actual Corona pandemic was according to me ‘misused’ to justify animal research. A large number of scientists (>100) had signed the letter and a couple of them are also participating in projects focusing on alternative approaches. I was disappointed about the polarizing way this was done and the lack of nuance. TPI Utrecht tried to depolarise the discussion and sent several nuanced letters to De Volkskrant but they did not get published, even without any response. I had the impression that they (researchers engaged mostly in animal research) were under pressure and I felt attacked too, because it was suggested that we (researchers developing animal-free methods) would paint everything too rosy. However, I thought the only way forward is to keep in contact and to connect people, in line with what we envision in our VHP4Safety project: the transition toward animal-free methods should involve all stakeholders, including the stakeholders that are less positive of the new approaches. I learned that my personal drivers can help me to connect with people that just like me are highly engaged and committed to contribute to human relevant developments in science and society.

## Discussion: exploring common ground on animal (−free) experimentation

After our individual experiences with the media, the ‘animal researcher’, Homberg, decided to apply for a Dutch communication project application, with the aim to improve the communication about animal research. She decided to do so with her colleagues Lisa Genzel (Associate professor, Radboud University, working with mice and rats) and Monique Wolvekamp (Senior Advisor Animal Ethics and Outreach, Radboud University, working with mice, rats, hamsters, dogs and pigs in the past). The driving factor was the perceived lack of knowledge in society and among various stakeholders about why and how animal research is being done, about alternative methods, as well as that science is about choosing the right method for the specific research question at hand and not about the right or wrong of methods *per se*. Homberg, Genzel and Wolvekamp felt the need to provide the public with honest, transparent information about animal research, allowing the public to form an opinion about animal research themselves. This would be in alignment with the Dutch transparency agreement for animal research, in which 20 institutions commit themselves to open transparent communication about animal research. During their first attempt they wrote the proposal from their perspective and proposed to have meetings with stakeholders for fact-based neutral communication about scientific methods. Unfortunately, and perhaps not surprisingly, the proposal was rejected, in part because they presented the proposal from their view as ‘animal researchers’ only.

To improve the proposal, during the next grant writing attempt they reached out to ‘animal-free innovation researchers’ Aldert Piersma and Cyrille Krul as well as Anne Kienhuis (Senior Scientist animal-free innovations in toxicology, who started her career comparing *in vivo* models (rats) with *in vitro* models (primary rat hepatocytes) to predict human toxicity) from the start. The idea was that valuable discussions amongst all stakeholders would allow improvement of the framing of the proposal, such that the proposal covered a joint need for improved communication and transparency about research methods, as is also key in the tool BATI (Beyond Animal Testing Index) developed by Krul (4). When the animal researchers Homberg, Genzel, and Wolvekamp approached the animal-free innovation researchers Piersma, Krul, and Kienhuis, to join the NWA communication project proposal. Though the proposal was rejected again, the reviewer score had significantly improved. In our perception, the rejection was partly due to a certain naiveté on our part as to the complexity of the issue and how to best address it. This resulted in the eye-opener from our side that we first needed to explore our joint ambition to improve the communication around animal research and animal free methods, among scientists that work either with animal or animal-free methods, before we would be able to communicate to a larger stakeholder group. This was also Based on funding from the Donders Institute for Brain, Cognition, and Behaviour, we organized a closed retreat for the animal researchers and animal-free innovation researchers involved, led by an independent moderator (Ellen ter Gast, who worked a long time ago with rats and goldfish and has been chair of an animal experimentation committee).

During the retreat, we first of all clarified that we are working in different research fields. Homberg and Genzel are working in the field of Neuroscience, which focuses on understanding the brain mechanisms underlying behavior and cognition. The brain is the most complex organ of the body and thereby difficult to study using non-living methods. On the other hand, animal experimentation in brain research is dependent on the brain function that is being studied, as evolutionarily conserved functions can (e.g., responses to threat, memory), while functions that make humans unique (e.g., complex language) cannot be studied readily in animals.

For Piersma, Krul, and Kienhuis the innovation of methods for chemical and pharmaceutical safety and risk assessment has been the focus of decades of research. This stems from the growing realization that current regulatory animal study protocols have limitations as to their extrapolation to humans. Moreover, new methods in molecular sciences, cell culture and computational methods have opened new and promising avenues toward a more human focused approach to hazard and risk assessment. Piersma, Krul and Kienhuis apply these new approaches in the lab, in close interaction with experts in the regulatory field, in order to facilitate eventual implementation in international legislation.

When meeting each other in person, we established that in spite of the contrasts between opinions of research groups suggested in the media broadcasts, we have common ground on principal issues related to animal experimentation. We quickly learned that our research fields and questions are very different and that discussion on the applicability of methods should always be considered within a specific context of use. Therefore, direct comparison of the methods we are using in our different practices is not possible. We also learned that we are all intending to identify and use the models best suited to address our research questions. Thus, asking the relevant research questions comes first, followed by the identification of the best available models to address those questions. This requires an open mindset, so that research questions are not formulated as tailored to an *a priori* preferred method (hobby horse), but actively allowing for alternative methods to be considered. This notion is equally relevant for researchers using non-animal models as for those using animal models. At the same time, as was earlier stated by George Box (5), we do realize that “all models are wrong, but some are useful.” We all use models with the aim to obtain human-relevant biomedical or safety-related information, with the awareness that the models we use have their specific advantages and disadvantages. This is true for all models. Animal models allow investigation of biological processes in an intact organism with interacting organs. At the same time, they are criticized for their low translatability to humans. For instance, it is often said that only 10% of all animal experiments lead to medicines that can be used by humans. Non-animal models make use of human cells or data with the promise to increase relevance and therefore translatability to humans. However, non-animal models still lack the ability to fully represent an intact organism and their translatability to humans, e.g., to contribute to effective and safe drug development, remains to be demonstrated. Hence, while we were presented as each other’s opponents by the media, we actually have fundamental common ground: striving for the best science possible, with methods fit to the purpose of the research, and awareness of the limitations of the models we use. This includes what has been termed the nine key characteristics of good science: objectivity, verifiability, ethical neutrality, systematic exploration, reliability, precision, abstraction and predictability (6). In the current context of *in vivo* experimentation, we define the ethical aspect of scientific research as a continuous search for optimal balance by thorough weighing of the pros and cons of animal use, including possibilities for Replacement, Reduction, and Refinement. The use of animal-free innovations directly contributes to Replacement and indirectly may also contribute to Reduction. That is, the use of animal-free innovation may reduce animal usage, although sometimes findings obtained via the use of animal-free methods still need to be verified using animal models. Simultaneously, animal researchers focus on Refinement, by technical advancements to get more info from a single animal and reducing animal discomfort as much as possible and thereby also reducing animal usage. This is important, because good science requires optimal animal housing. This is amongst others achieved by providing the animals more space and enrichment to fulfill their needs according to the context in which they originally evolved.

Establishing common ground also created the awareness that we can improve communication on animal(−free) experimentation issues by teaming up. By being able to view our own communication through the eyes of colleagues who use a different set of research methods, we are better able to convey our messages, reducing triggers for emotional responses. This interaction has emphasized the importance of placing our own communication in the context of that of the various stakeholders involved in animal research.

### Generic lessons learned

Below we summarize our five generic lessons learned:

1. The principles of good science provide the fundamental basis for selecting research models and methods
2. Careful formulation of the research questions precedes, informs and determines the choice of the optimal models to answer those questions. This requires an open mindset, so that research questions are not formulated tailored to an *a priori* defined method, but actively allowing for alternative methods to be considered.
3. Fruitful communication between scientists on animal(−free) experimentation involves the leans on a factual basis, combined with the willingness to consider different perspectives. An open dialog is needed realizing the differences in research fields requiring different methodologies. From a scientific perspective, these may include animal-free methods when possible and animal methods when still needed, as are best fit to the research questions at hand.
4. The multi-stakeholder debate on animal experimentation is intrinsically complex, given different perspectives, interests, dependencies, and beliefs. Scientific, ethical and regulatory considerations may provide opposing argumentation. The debate would gain from a transparent positioning of stakeholders and an open mindset as to mutual commonalities and differences.
5. The interaction of scientists with the media on animal (−free) research is important as it provides mutually advantageous opportunities. It provides scientists with an important platform for communication with the general public, whilst the media are provided with content for broadcasting on a highly debated subject in society.

In conclusion, we have learned that, in spite of the media framing placing us in opposite positions in the discussion on animal research and animal-free innovations, we have significant common ground on the principles of good scientific research and responsible experimentation. Our communication emanating from the broadcasts has been instrumental in realizing again the need for making well-informed decisions based on different perspectives for the methods we are using now and will be using in the future. We hope that our experiences will benefit other stakeholders involved in the debate on animal research in science.

## Data availability statement

The original contributions presented in the study are included in the article/supplementary material, further inquiries can be directed to the corresponding author.

## Author contributions

JH: Writing – original draft, Writing – review & editing. CK: Writing – original draft, Writing – review & editing. AP: Writing – original draft, Writing – review & editing. AK: Writing – original draft, Writing – review & editing. LG: Writing – original draft, Writing – review & editing. MW: Writing – original draft, Writing – review & editing. EG: Writing – original draft, Writing – review & editing.
